# Kawasaki shock syndrome in an Arab female: case report of a rare manifestation and review of literature

**DOI:** 10.1186/s12887-019-1662-9

**Published:** 2019-08-23

**Authors:** Ahmed A. Nugud, Assmaa Nugud, Deena Wafadari, Walid Abuhammour

**Affiliations:** 1 Aljalila Children’s specialty hospital, Dubai Health Care City, Dubai, UAE; 2 Sharjah institute for Medical Research, Sharjah, UAE. PoBox, 7226 Dubai, UAE; 3 RAK Medical & Health Sciences University, Ras al Khaimah, UAE

**Keywords:** Kawasaki disease, Shock, Kawasaki disease shock syndrome

## Abstract

**Background:**

Kawasaki disease shock syndrome is a relatively new and rare complication of Kawasaki disease first described in 2009.

**Case presentation:**

This report describes a two-year-old Arab descent female presenting with a history of high-grade fever of 2 days duration with non-specific signs of viral illness and erythematous rash. The patients’ condition deteriorated rapidly requiring admission to intensive care unit. In the intensive care unit, she developed a right upper quadrant mass that was diagnosed as hydrops of the gallbladder by ultrasonography. After one dose of intravenous immunoglobulin, the patient started to recover and was transferred out of intensive care after 2 days.

**Conclusion:**

Among the complications of Kawasaki disease, shock syndrome is rare and usually will have deleterious results if not diagnosed and managed promptly.

**Electronic supplementary material:**

The online version of this article (10.1186/s12887-019-1662-9) contains supplementary material, which is available to authorized users.

## Background

Also known as mucocutaneous lymph node syndrome, KD was first described in 1967 by Dr. Tomisaku Kawasaki [1]. KD is a medium sized vessel systemic vasculitis of an unknown etiology [2]. KD prevalence is higher in Asian countries in comparison to Western countries. The highest incidence rate is seen in Japan followed by Korea and Taiwan, while lower rates are seen in Europe. A well-established hypothesis about the cause of KD incriminates infectious agents as triggers to an inflammatory response, causing a dysregulation in the host-immune reaction in genetically predisposed individuals [3]. An underlying genetic predisposition of KD is supported by studies that found a higher risk of KD in siblings of patients diagnosed with KD [4]. The most serious complication of KD is the development of a coronary artery lesion which is usually seen during the subacute phase [3]. KD is also recognized as the leading cause of acquired heart disease in children in the United States [4].

A small subset of KD patients do not meet the classical presentation of KD and thus are termed incomplete KD. Incomplete KD patients are usually infants and older children and are at a higher risk of developing cardiac lesions [4]. Hemodynamic instability during the acute phase of KD is an uncommon manifestation of KD. Kanegaye et al., in 2009, identified and described the term KD shock syndrome (KDSS) [5]. The cause of severe hypotension in KDSS is unknown, but it is believed to be due to the ongoing vasculitis complicated with capillary leakage, myocardial dysfunction, and generalized cytokine dysregulation [5].

## Case presentation

A two-year-old Arab female presented to the Emergency Department (ED) with a 2 day history of high-grade fever up to 40C^o^, cough and rhinorrhea. On the third day of illness, she developed dehydration secondary to vomiting and severe watery diarrhea requiring admission for intravenous (IV) fluid rehydration. On the fourth day of fever, she developed an erythematous rash over the face and trunk. A rapid respiratory viral panel was positive for Influenza B virus.

On admission, she was alert but tired. Vital signs revealed fever at 38.8 C^o^, respiratory rate of 31 breaths per minute, heart rate of 150 beats per minute, blood pressure of 96/54 mmHg, with normal Oxygen saturation and capillary refill time. She had an erythematous rash over the face and the trunk, and cracked lips. Small bilateral submandibular lymph nodes were palpable; throat was congested with hyperemic tonsils. The remainder of the physical examination was unremarkable.

After 10 h of admission, she developed lethargy and hypotension with BP of 78/41 mmHg. Cold extremities and delayed capillary refill time, facial puffiness with swollen hands and feet were noted. Abdomen examination revealed right upper quadrant distension, and was otherwise unremarkable with no hepatosplenomegaly. As she was manifesting symptoms of shock and deteriorating clinical status, blood cultures were obtained along with further investigations (Table 1), IV Ceftriaxone was initiated as the primary physician suspected septic shock, and she was transferred to the Pediatric Intensive Care Unit for further observation and management. Antibiotic therapy was stopped after the blood culture result returned negative.

**Table 1:** Significant laboratory finding on admission and after 10 h of admission

	IN Emergency Department	10 h post admission
WBC	8.16 × 10 [3]/mcl (N 67%, L 28%)	6.29 × 10 [3]/mcl (N 69.4%, L 21%)
HB	11.50 g\l	9.1 g\l
Platelets	104 × 10 [3]/mcl	87 × 10 [3]/mcl
CRP	24 mg/dl	50 mg/dl
ESR	–	13 mm
Total protein	–	4.74 g/dl (Albumin 2.8)
Procalcitonin		10 ng/ml
Virology	Positive for Influenza B Virus	

A diagnosis of KDSS was considered after consultation to the pediatric infectious disease doctor, and therefore Intravenous Immunoglobulin (IVIG) at a dose of 2 g/kg was given. Aspirin was deferred as she was influenza B positive. An ultrasound of the abdomen was obtained which revealed hydrops of the gallbladder. Initial echocardiography (ECHO) was normal. After 24 h of the IVIG therapy, the patient was afebrile, and her general condition started to improve. Two days after IVIG, she developed peeling mainly at the neck and perianal area. She was discharged from the hospital in good condition and follow up ECHOs were normal.

## Discussion and conclusion

Given the overlapping initial clinical presentation between KDSS and TSS, some experts believe that superantigen (SAg) mediated inflammation could be the underlying mechanism of both KDSS and TSS. The findings of Nagata et al., in 2009, support this hypothesis; in which the authors found the Sag producing microorganisms in the gastrointestinal tract of KD patients [6]. Many pathogens have been linked to the pathogensis of KD, including Influenza virus, like in the case at hand. Many reports through the literature reported KD after Influenza (A and/or B) infection; interestingly enough Shimada et al. in 15 reported a case of KD following influenza vaccine [7].

Cardiac complications of untreated or delayed treatment of KDSS include coronary artery dilation and aneurysmal formation, valvular regurgitation, and persistent ventricular diastolic dysfunction^5,^ Gámez-González et al., in 2018 reported coronary arteries abnormalities in 72% of in a sample of 103 patients with KDSS [8]. Gastrointestinal symptoms of KDSS include abdominal pain, vomiting, diarrhea and gastrointestinal bleeding, as reported by Gámez-González et al.[9].

Studies have found that KDSS patients had lower age-adjusted hemoglobin z score, lower serum albumin levels, bandemia, neutrophilia, thrombocytopenia, and higher CRP levels when compared to KD patients [5]. Although KD is commonly seen in males, KDSS patients are predominantly females [10]. The cause of severe hypotension seen in KDSS remains unknown. A common hypothesis explains in relation to contractile dysfunction of myocytes resulting from massive cytokine production, leading to the development of systemic capillary leak syndrome due to elevated interleukin-2 levels and increased endothelial cell permeability [11, 12].

Chen et al. in 2013 found that KDSS patients had a delayed diagnosis in comparison to KD patients, longer hospital stay by 9.2 days on average, and a higher rate of failure after the first IVIG treatment. This was not reflected in this case. In addition, KDSS patients had higher levels of inflammatory markers. The authors attributed these findings to delayed diagnosis and treatment initiation in KDSS^**Error! Bookmark not defined.**^. While Ma et al., in 2017, found that KDSS patients had higher levels of liver enzymes, procalcitonin, brain natriuretic peptide, troponin I, and ferroprotein [13].

This case highlights a rare but severe complication of KD. Recognition and vigilance for the manifestation of KDSS is critical for timely treatment which can have a significant impact on the outcome. To our knowledge, this is the first report of KDSS in the Arab region. Since KDSS was first reported in 2009, many retrospective studies were carried out that are not included in our review. To the best of our knowledge the incidence of KD/KDSS is not known in the region as there has been limited data reported from the Middle East. Another reason is the lack on census data in the region for children below 5 years of age [14]. A recent case report from the region showed KD presenting with findings of acute renal failure, thrombocytopenia, hemolytic anemia and gastrointestinal enteritis [15]. Additional file 1: Table S1 summarizes the clinical findings in all prospective published cases since 2009.

## Additional file

Additional file 1: **Table S1.** Demographic and clinical data of all prospective published KDSS studies.

## Data Availability

Not applicable.

## Abbreviations

BCG: Bacillus Calmette–Guérin
CXR: chest X-ray
EBV: Epstein-Barr viral infection
ECHO: Echocardiography
ED: Emergency Department
FUO: Fever of unknown origin
ICU: Intensive Care Unit
IKD: Incomplete Kawasaki disease
IV: Intravenous
IVIG: Intravenous Immunoglobulin
KD: Kawasaki disease
KDSS: Kawasaki Disease Shock Syndrome
LFT: Liver function test
M: month
N/A: Data not available/ or not applicable
RS: Respiratory distress
SAg: Superantigen
Y: year
