# Supplementary material for: Kawasaki shock syndrome in an Arab female: case report of a rare manifestation and review of literature
Source: BMC Pediatr. 2019 Aug 23;19:295. doi: 10.1186/s12887-019-1662-9 (PMC6706932; doi:10.1186/s12887-019-1662-9)
Supplement: Supplementary file 1 — Table S1. Demographic and clinical data of all prospective published KDSS studies. [file 12887_2019_1662_MOESM1_ESM.docx]

Table S1: Demographic and clinical data of all prospective published KDSS studies.

| Age | Gender | | Signs and symptoms | | | | | ECHO findings | ICU transfer reason | Reference |
| --- | --- | --- | --- | --- | --- | --- | --- | --- | --- | --- |
|  | M | F | Mucocutaneous manifestations:  A- Rash  B- BCG erythema  C-Desquamation  D-Lip cracking  E- Strawberry tounge  F- Conjunctivitis | Pulmonary | Gallbladder Hydrops | Cervical lymphadenopathy | Others |  |  |  |
| 1 Y | ✔ |  | A, D, E, F | N/A | N/A | ✔ | Fever for 11 days | N/A | Sepsis | Chen at al^2^ |
| 1M |  | ✔ | A, B, C, D, E | RS |  |  | Fever for 15 days, Seizures at presentation | N/A | RS and seizures | Chen at al^2^ |
| 4M | ✔ |  | A, B, C, D, E, F | N/A | N/A | N/A | Fever for 8 days | N/A | Sepsis | Chen at al^2^ |
| 3Y 9M |  | ✔ | A, C, D, E, F | N/A | N/A | ✔ | Fever for 5 days | N/A | Hypotensive shock | Chen at al^2^ |
| 7Y |  | ✔ | A, D, E, F | N/A | N/A | N/A | Fever for 7 days | N/A | Septic shock | Chen at al^2^ |
| 9 M |  | ✔ | A, D, E, F | N/A | N/A | N/A | Fever for 8 days, presented with acute gastroenterocolitis | N/A | Hypotensive shock | Chen at al^2^ |
| 10 Y | ✔ |  | A, F | N/A | N/A | ✔ | Fever for 10 days, miss diagnosed as toxic erythema initially | N/A | Sepsis | Chen at al^2^ |
| 2Y 7M |  | ✔ | D, E, F | RS | N/A | ✔ | Fever for 11 days patient also had retropharyngeal abscess | N/A | RS | Chen at al^2^ |
| 2Y 3M |  | ✔ | D, E, F | N/A | N/A | N/A | Fever for 6 days | N/A | Septic shock | Chen at al^2^ |
| 15 Y | ✔ |  | A, F | N/A | N/A | N/A | Fever for 4 days, Requiered two doses of IVIG, managed as case of acute abdomen initially | Left coronary artery hyperechogenicity,Left ventricular dysfunction | Left ventricular dysfunction | Marrani et al^[[1]](#endnote-1)^ |
| 7Y |  | ✔ | A, D, E, F | Bilateral interstial infiltrates on CXR | N/A | ✔ | Fever for 4 days, required 2 IVIG doses, Hepatomegaly 3cm below costal margin. | Mild mitral regurgitation, Left Anterior descending artery aneurysm | Septic shock | To et al^[[2]](#endnote-2)^ |
| 2Y 2M |  | ✔ | A, C, D, E, F | N/A | N/A | ✔ | Had generalized edema at presentation, fever for 6 days, Hepatomegaly 3cm below costal margin, required 2 IVIG doses and 3 day course of high dose methylprednisolone | Mild mitral regurgitation, Left coronary artery dilatation, Mild left atrial and ventricular dilatation | N/A | To et al^13^ |
| 11Y | ✔ |  | C, F | N/A | ✔ | ✔ | Fever for 6 days, had sever myalgia | Mild mitral regurgitation, LAD dilatation, required 2 IVIG doses and 3 day course of high dose methylprednisolone | Hypotensive shock | Cakan et al^[[3]](#endnote-3)^ |
| 8Y |  | ✔ | A, C, F | Pulmonary edema, Plural effusion and RS | N/A | N/A | Fever for 3 days, required 2 doses of IVIG, had hepatomegaly with increased LFT, rash resembled scarlet fever rash | Normal Echo | Toxic shock syndrome | Cakan et al^14^ |
| 2Y 2M | ✔ |  | A, D, E, F | N/A | N/A | ✔ | Fever for 8 days | N/A | Sepsis | Ma et al^10^ |
| 13Y | ✔ |  | A, D, E, F | N/A | N/A | ✔ | Fever for 5 days, Palmar erythema | N/A | N/A | Ma et al^10^ |
| 6Y 2M | ✔ |  | A, F | N/A | N/A | ✔ | Fever for 9 days, in additon had parotitis | N/A | Septic shock | Ma et al^10^ |
| 1M |  | ✔ | A | N/A | N/A | N/A | Fever for 15 days | N/A | FUO | Ma et al^10^ |
| 6Y 5M |  | ✔ | A, B, F | N/A | N/A | ✔ | Fever for 8 days, elevated LFT | N/A | Sepsis | Ma et al^10^ |
| 8Y 8M | ✔ |  | A, B, C, D, E, F | N/A | N/A | ✔ | Fever for 6 days | N/A | N/A | Ma et al^10^ |
| 3Y 8M |  | ✔ | A, B, C, D, E, F | N/A | N/A | ✔ | Fever for 6 days | N/A | N/A | Ma et al^10^ |
| 1Y 9M | ✔ |  | A, F | N/A | N/A | N/A | Fever for days | N/A | N/A | Ma et al^10^ |
| 6Y |  | ✔ | A, F | N/A | N/A | ✔ | Fever for 2 days, initially managed as EBV infection, presented with erythema annulare rash | N/A | N/A | Ma et al^10^ |
| 5Y 5M |  | ✔ | N/A | N/A | N/A | ✔ | Fever for 9 days, | N/A | Hemolytic anemia and sepsis | Ma et al^10^ |
| 4Y 3M |  | ✔ | A, D, E, F | N/A | N/A | ✔ | Fever for 6 days, lymphadenitis on admission | N/A | N/A | Ma et al^10^ |
| 4Y 3M | ✔ |  | A, B, D, E, F | N/A | N/A | ✔ | Fever for 5 days, lymphadenitis on admission | N/A | N/A | Ma et al^10^ |
| 5Y 5M |  | ✔ | A, B, D, E, F | N/A | N/A | ✔ | Fever for 5 days | N/A | N/A | Ma et al^10^ |
| 4Y 1M |  | ✔ | A, D, E | N/A | N/A | ✔ | Fever for 5 days, initial diagnosis as IKD | N/A | N/A | Ma et al^10^ |
| 1Y 4M | ✔ |  | B, D, E, F | N/A | N/A | N/A | Fever for 5 days | N/A | Sepsis | Ma et al^10^ |
| 5Y 7M | ✔ |  | A, B, C, D, E, F | N/A | N/A | ✔ | Fever for 6 days | N/A | N/A | Ma et al^10^ |
| 4Y 5M | ✔ |  | A, B, C, D, E, F | N/A | N/A | ✔ | Fever for 6 days | N/A | N/A | Ma et al^10^ |
| 2Y 7M |  | ✔ | A, B, C, D, E, F | N/A | N/A | ✔ | Fever for 5 days, erythema of the soles and palms | N/A | N/A | Ma et al^10^ |
| 6Y 1M | ✔ |  | A, B, C, D, E, F | N/A | N/A | ✔ | Fever for 5 days, erythema of the soles and palms | N/A | N/A | Ma et al^10^ |
| 2Y 4M |  | ✔ | A, B, C, D, E, F | N/A | N/A | ✔ | Fever for 5 days | N/A | N/A | Ma et al^10^ |
| 6Y 9M | ✔ |  | A, B, C, D, E, F | N/A | N/A | ✔ | Fever for 7 days, erythema of the soles and palms | N/A | N/A | Ma et al^10^ |
| 12Y |  | ✔ | A, F | Pleural effusion | N/A | ✔ | Fever for 3 days, had periorbital edema on presentation, developed mild ascites without hepatosplenomegaly, initially managed as case of dengue fever | Left anterior descending artery dilatation | Hypotensive shock | Sinhabahu et al^[[4]](#endnote-4)^ |
| 10Y | ✔ |  | A, D, E, F | N/A | ✔ | N/A | Fever for 6 days; presented with intermittent confusion, headachs, vomiting and myalgia, had maculopapular rash with target lesions on presentation, labs showed renal impairment and raised liver enzymes, Rhinovirus positive on nasal swab | pericardial effusion and biventricular systolic  dysfunction, generalized dilatation and loss of  normal tapering of the right coronary artery | hemodynamic  instability | Yim et al^[[5]](#endnote-5)^ |
| 12Y | ✔ |  | A, F | N/A | N/A | N/A | Fever for 5 days, presented with vomiting and headaches; widespread target  lesions over his trunk and extremities, and punctate  ulcers on his buccal mucosa, Abdominal examination revealed right  upper-quadrant abdominal tenderness. Required two doses of IVIG. | biventricular dysfunction with a fractional shortening, moderate tricuspid regurgitation, and  diffuse dilatation of the left main coronary artery.  Developed first-degree heart block and an  intermittent junctional rhythm after IVIG treatment. | hemodynamic instability | Yim et al^15^ |
| 10Y |  | ✔ | All negative | N/A | N/A | N/A | Fever for 2 days; Dengue antibody was negative; after one does of IVIG she developed peeling of skin  during the 2nd week of illness | uniformly  dilated right and left coronary arteries, with an  ejection fraction of 55%. | N/A | Sathiadas et al^[[6]](#endnote-6)^ |
| 11Y | ✔ |  | A, B, C, D, E, F | N/A | N/A | N/A | Fever and throat pain for 4 days; dengue antibody was negative; improved after one dose of IVIG | left anterior descending and right  coronary artery, a thin rim of pericardial effusion and  an ejection fraction of 50%. | Hypotensive shock | Sathiadas et al^16^ |
| 4Y | ✔ |  | A, E, F | N/A | N/A | N/A | Fever and generalized rash for 6 days; There was a 4 cm tender hepatomegaly; had elevated liver enzymes and dengue antigen and  antibody were negative. | mildly reduced left  ventricular function, thin rim of pericardial effusion,  uniformly dilated right and left (4mm both)  coronary arteries and no aneurysms of the coronaries | Developed circulatory shock on the second day of admission | Sathiadas et al^16^ |

1. Marrani E, Giani T, Paganelli V, Simonini G, Pagnini I, Calabri G et al. Kawasaki shock syndrome: a case report. Pediatric Rheumatology. 2014;12(S1). [↑](#endnote-ref-1)
2. To, S., Yan, C., Fong, N., & Leung, C. Two Cases of Kawasaki Disease Shock Syndrome. Hong Kong Journal Of Pediatrics, 2016;21, 197-200. [↑](#endnote-ref-2)
3. Çakan M, Gemici H, Aktay-Ayaz N, Keskindemirci G, Bornaun H, İkizoğlu T et al. Kawasaki disease shock syndrome: a rare and severe complication of kawasaki disease. The Turkish Journal of Pediatrics. 2016;58(4):415. [↑](#endnote-ref-3)
4. Sinhabahu V, Suntharesan J, Wijesekara D. Kawasaki Shock Syndrome in a 12-Year-Old Girl Mimicking Septic Shock. Case Reports in Infectious Diseases. 2016;2016:1-3. [↑](#endnote-ref-4)
5. Yim D, Ramsay J, Kothari D, Burgner D. Coronary Artery Dilatation in Toxic Shock-Like Syndrome: The Kawasaki Disease Shock Syndrome. Pediatric Cardiology. 2010;31(8):1232-1235. [↑](#endnote-ref-5)
6. Sathiadas M, Umashankar N, Ragunathan R, Thangarajah K. Kawasaki disease shock syndrome: three cases. 2019.

   **Figure legends:**

   Figure 1: Right upper quadrant ultrasound showing hydrops of the gallbladder. [↑](#endnote-ref-6)
